# A comprehensive analysis of *MAPT*-related genetic risk in Alzheimer’s disease

**DOI:** 10.1016/j.ibneur.2025.01.017

**Published:** 2025-02-03

**Authors:** Shitao Wang, Guoshuai Luo, Xiangqian Ding, Guangxin Sun, Mengen Zhang, Jingjing Dong, Hui Xu, Jinghong Lu, Zongyou Li, Bin Ning, Hongbo Liu

**Affiliations:** a Department of Neurology, Affiliated Fuyang People's Hospital of Anhui Medical University, Fuyang, Anhui, China; b Laboratory of Biological Psychiatry, Institute of Mental Health, Tianjin Anding Hospital, Mental Health Center of Tianjin Medical University, Tianjin, China; c Department of neurosurgery, Qilu Hospital of Shandong University, Jinan, Shandong, China; d Department of Cardiovascular Medicine, Affiliated Fuyang People's Hospital of Anhui Medical University, Fuyang, Anhui, China; e Department of Emergency, Affiliated Fuyang People's Hospital of Anhui Medical University, Fuyang, Anhui, China

**Keywords:** Alzheimer’s disease, Rs2471738, MAPT, Tau, Polymorphism

## Abstract

Despite some research into the correlation between microtubule associated protein tau (*MAPT*) rs2471738 and the risk of AD, the findings remain inconclusive. The aim of this study was to explore the association between *MAPT* rs2471738 and the susceptibility to AD, as well as potential molecular mechanisms involved. We conducted a comprehensive literature search on Embase, Medline, and Web of Science to investigate the relationship between *MAPT* rs2471738 and the risk of AD. We employed meta-analysis and Expression Quantitative Trait Loci analysis to elucidate the association between *MAPT* rs2471738 and AD risk, as well as to uncover potential molecular mechanisms. Aggregated results suggest that the rs2471738T allele increases the risk of developing AD under the allelic model (odds ratio [OR] = 1.15, 95 % confidence interval [CI] = 1.04–1.26, I² = 64.9 %). Additionally, our findings indicate that the rs2471738CT+TT genotype escalates the risk of AD under the dominant model (OR = 1.23, 95 % CI = 1.07–1.41, I² = 79.2 %). Moreover, rs2471738 regulates the expression of *MAPT* in the human hippocampus (*P* = 0.04). Our result suggested that rs2471738 may potentially increase the risk of AD by modulating the expression of *MAPT* in human brain tissue.

## Introduction

Alzheimer’s disease (AD) is characterized by the pathological accumulation of Aβ and the formation of intraneuronal neurofibrillary tangles, which result from the aggregation of hyperphosphorylated microtubule associated protein tau (MAPT) (Scheltens et al., 2016). Notably, these neurofibrillary tangles, composed of MAPT aggregates, play a pivotal role in the pathogenesis of AD (Bakota and Brandt, 2016). In the context of AD, MAPT undergoes hyperphosphorylation, leading to the formation of insoluble aggregates. Consequently, this impairs the ability to maintain microtubule stability (Medeiros et al., 2011). In addition to phosphorylation, acetylated MAPT has been demonstrated to contribute significantly to MAPT-mediated synaptic toxicity (Tracy et al., 2016, Min et al., 2015). Furthermore, research has indicated that the interaction between soluble Aβ and MAPT plays a crucial role in AD (Bloom, 2014), Busche and Hyman, 2020).

*MAPT*, which encodes the tau protein, is located on chromosome 17. This locus has been linked to an increased risk of AD (Jun et al., 2016). The rs2471738 polymorphism, found within the *MAPT* intron, has been previously associated with AD risk (Myers et al., 2007, Myers et al., 2005, Mateo et al., 2008, Vázquez-Higuera et al., 2009). However, conflicting results have been reported in other studies (Cousin et al., 2011, Abraham et al., 2009, Allen et al., 2014). The mechanism by which rs2471738 increases the risk of AD remains unclear. Therefore, further evaluation of the relationship between rs2471738 and the risk of developing AD is necessary.

In this study, we incorporated a large sample from high-quality studies, consisting of 12,750 AD patients and 15,868 healthy controls, to investigate the association between rs2471738 and the risk of developing AD. Furthermore, to elucidate the potential molecular mechanism of rs2471738 in the pathogenesis of AD, we conducted an Expression Quantitative Trait Loci (eQTL) analysis using human brain tissue to explore the relationship between rs2471738 and the expression of *MAPT*.

## Experimental methods

### Literature search

We systematically searched Embase, Medline, and Web of Science for studies on the association between *MAPT* rs2471738 and the risk of AD. The search, conducted up to December 31, 2024, employed the following terms: (*MAPT* OR tau) AND (polymorphism OR variant) AND (Alzheimer’s disease). No language restrictions were imposed during the literature search.

### Inclusion and exclusion criteria

The criteria for inclusion were as follows: (1) All subjects reported genotype data, and (2) The research focused on AD. Documents that meet the following criteria are excluded: (1) Genotype could not be obtained, (2) The review was excluded but its references were used to identify relevant studies, (3) Papers that were only abstracts, did not relate to the relationship between rs2471738 and AD, lacked available genotype data, were animal studies, or were cell studies, and (4) The genotype distribution in the control group does not conform to Hardy-Weinberg equilibrium (HWE).

### Data extraction and quality assessment

Three investigators, Guangxin Sun, Hongbo Liu and Mengen Zhang reviewed and extracted data from the literature in accordance with the inclusion criteria. If there is a disagreement, Jinghong Lu will solve the problem arising from the disagreement. Xiangqian Ding and Jingjing Dong independently assessed the quality of the included literature using the Newcastle-Ottawa Scale (Stang, 2010). Studies scoring six points or above were considered to be of high quality.

### Statistical analysis

The statistical analyses were conducted using STATA software edition11.0 (Stata-Corp LP, College Station, TX, U.S.A.) and SPSS software 23.0(IBM Corp, Armonk, NY). The HWE in control groups was calculated using the Chi-square test. Significant heterogeneity was considered if the p-value was less than 0.05 or I^2^ exceeded 50 %. In terms of heterogeneity, if there is no significant heterogeneity, a fixed-effects model is used for analysis; otherwise, a random-effects model is used for analysis. In order to assess the sensitivity of this study, a method involves gradually excluding each study included in the research and then evaluating the changes in ORs and 95 % CIs for the studies that were not excluded. Additionally, Egger’s test (Egger et al., 1997) was conducted to evaluate the potential publication bias, with a p-value of less than 0.05 indicating significance.

### Expression quantitative trait loci (eQTL) analysis

The Genotype-Tissue Expression Portal (GTEx, http://www.gtexportal.org/ home/) (GTEx Consortium, 2013) serves as a comprehensive public resource for researchers who are investigating tissue and cell-specific gene expression and regulation across individuals, developmental stages, and species. It includes data from three NIH projects. In this study, we utilized eQTL data from brain tissues available in the GTEx database to assess whether rs2471738 regulates the expression levels of *MAPT* in human brain tissue.

## Results

### Study selection

**Fig. 1:**
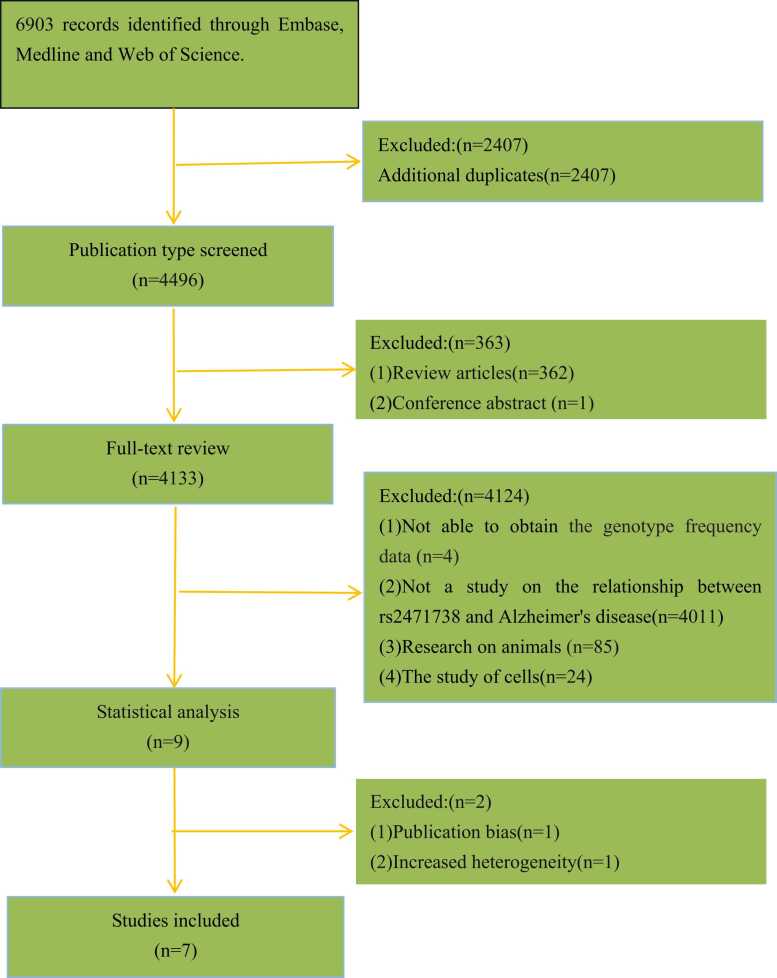
Flow diagram of selection of studies.

As shown in Fig. 1, after reviewing the publication types and examining the full texts, a total of 6894 studies were excluded based on the inclusion and exclusion criteria. Additionally, we conducted quality, statistical, and HWE assessments for the studies considered for inclusion. Ultimately, seven high-quality studies, involving a total of 28,618 participants, were included in the analysis. The key characteristics of these seven studies are summarized in Table 1.

**Table 1:** Characteristics of included studies.

**Author**	**Year**	**Origin**	**AD/Control**	**HWEp**	**NOS score**
Cousin et al.	2011	France	423/454	0.982	7
Abraham et al.	2009	UK	970/1125	0.586	8
Allen(Mayo Cohort) et al.	2014	USA	1980/3302	0.313	8
Allen(ADGC Cohort) et al.	2014	USA	6942/7239	0.927	8
Allen(RS) et al.	2014	USA	585/2355	0.753	8
Mateo et al.	2008	Spain	293/396	0.618	8
Myers(US) et al.	2005	USA	181/131	0.898	8
Myers(UK) et al.	2005	UK	179/121	1.000	8
Myers(US) et al.	2007	USA	296/128	0.343	8
Myers(US+UK) et al.	2007	USA+UK	655/380	0.937	8
Vazquez-Higuera et al.	2009	Spanish	246/237	0.813	8

NOS, the Newcastle-Ottawa Scale; AD, Alzheimer’s Disease; HWE, Hardy-Weinberg Equilibrium in controls.

### Association of Rs2471738 polymorphism with the risk of AD

**Fig. 2:**
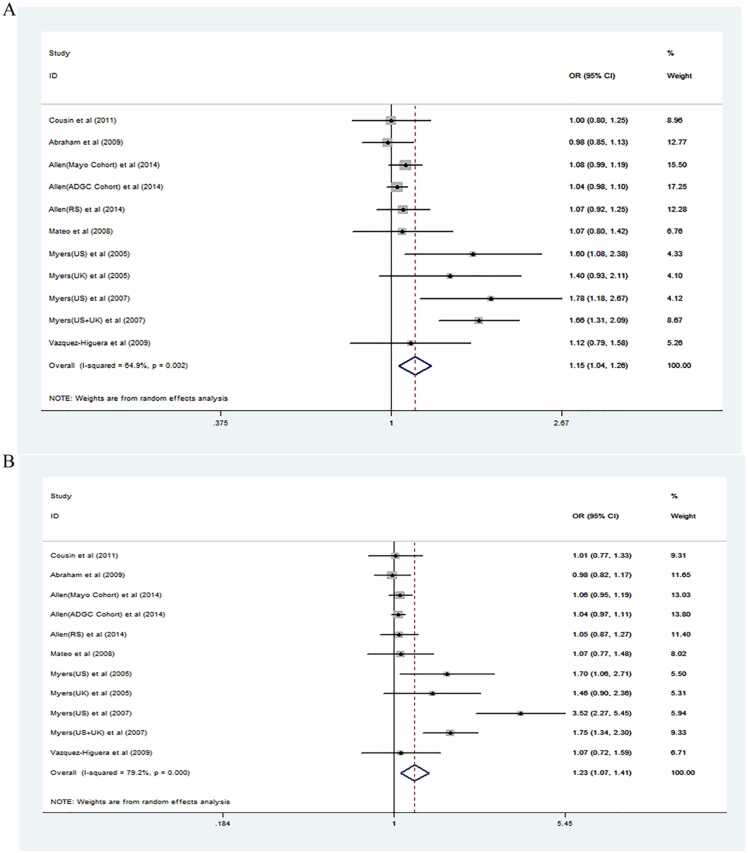
Forest plots showing the associations between rs2471738 and Alzheimer's disease. (A) Based on the Tvs.C model. (B) Based on the TT+CT vs.CC model.

A comprehensive study was conducted on a total of 28,618 subjects to evaluate the association between the rs2471738 polymorphism and the risk of AD. The pooled results suggested that the presence of the rs2471738T allele escalates the risk of developing AD under the allelic model (OR = 1.15, 95 %CI = 1.04–1.26, I² = 64.9 %) as depicted in Fig. 2A. Furthermore, it showed that the rs2471738CT+TT genotype amplified AD risk under the dominant model (OR = 1.23, 95 % CI = 1.07–1.41, I² = 79.2 %), as shown in Fig. 2B.

### Publication bias and sensitivity analysis

**Fig. 3:**
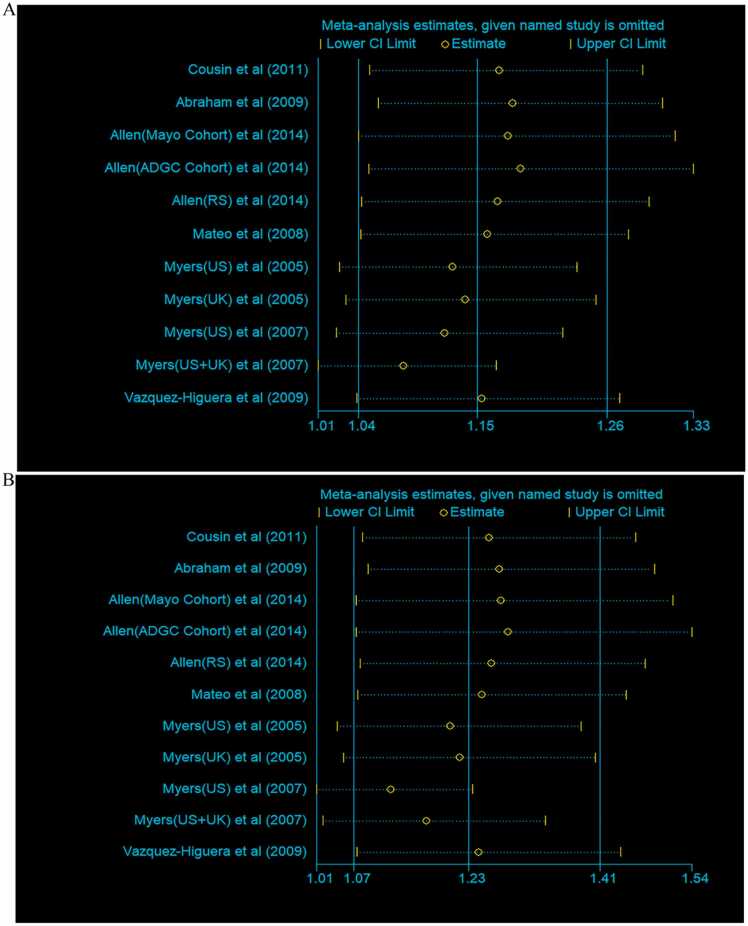
Sensitivity analysis of studies on the association between rs2471738 and Alzheimer's disease.(A) Based on the Tvs.C model. (B) Based on the TT+CT vs.CC model.

Egger’s test indicated a potential publication bias under the allelic model (*P* = 0.04). Consequently, a trim and fill analysis was conducted to identify the source of this bias. However, no evidence of publication bias was found. Under the dominant model, Egger’s test did not reveal any potential publication bias (*P* > 0.05). A sensitivity analysis was performed, and it was found that no single study significantly influenced the overall combined odds ratio, as shown in Fig. 3. This suggests that our results are relatively reliable and stable.

### Expression quantitative trait loci (eQTL) analysis

**Fig. 4:**
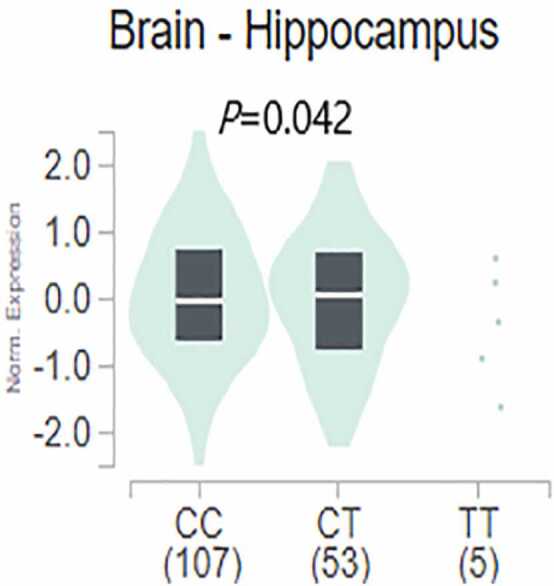
Regional regulation *MAPT* expression by rs2471738 genotype in human brain. Data were retrieved from the Genotype-Tissue Expression project (GTEx).

We analyzed eQTL data from 13 distinct brain regions using the GTEx database. Our analysis revealed that rs2471738 regulates MAPT expression in the human hippocampus (P = 0.04), as illustrated in Fig. 4. Additionally, Fig. 4 shows that the CT genotype carrying the T allele upregulates MAPT expression in the hippocampus compared to the CC genotype. However, due to the presence of only five TT genotype carriers in the hippocampus, the regulatory effect of the TT genotype on MAPT expression requires further investigation.

## Discussion

Aggregates of MAPT have been observed to induce hyperactivation of glial cells, leading to subsequent neuroinflammation. This process may also contribute to neurodegeneration in AD (Gulisano et al., 2018). Several other *MAPT* Single Nucleotide Polymorphisms (SNPs) have been associated with an increased risk of AD (Liu et al., 2013, Laws et al., 2007). The rs2471738, a SNP located in the intron region of *MAPT*, has been extensively studied for its relationship with the risk of AD. However, the conclusions drawn so far remain controversial. Therefore, it is imperative to further integrate data from large-scale, multi-center studies and simultaneously conduct functional analyses to elucidate the relationship between rs2471738 and the risk of AD.

In our study, we discovered that the rs2471738 polymorphism increases AD risk under both the allelic and dominant models. Specifically, the presence of T allele and CT+TT genotypes escalates the risk of developing AD. This observation aligns with a previous study conducted by Zhou et al. (Zhou and Wang, 2017), but contradicts the findings of Yuan et al. (2018). Upon reviewing the relevant literature, we noted significant variations in the sample sizes across different studies, which could be the primary reason for these inconsistent conclusions. Studies with larger sample sizes possess higher statistical power, enabling them to detect associations that might be overlooked in studies with smaller sample sizes. Our study, however, incorporated a large sample size, thereby ensuring high statistical power. Coupled with the stringent quality control measures we employed for the included literature, we were able to derive reliable conclusions.

The rs2471738 polymorphism is located in the intron region of *MAPT*. It’s often observed that SNPs in non-coding regions of genes can influence disease progression by regulating their target genes (Li et al., 2023, Li et al., 2023). The hippocampus, one of the brain areas significantly affected by AD (Rao et al., 2022), interestingly, is found to have its *MAPT* expression regulated by rs2471738 in our study. These findings shed light on the potential molecular mechanism of AD pathogenesis instigated by rs2471738.

This study has several strengths: (1) To clarify the potential mechanism of rs2471738 in AD, we explored the relationship between rs2471738 and *MAPT* expression in human brain tissue for the first time. (2) We rigorously controlled the quality of the included literature, ensuring the reliability of the conclusions. (3) We included a relatively large sample size with high statistical power, which is advantageous for identifying non-significant AD etiology. However, some limitations should also be acknowledged: (1) Due to the inclusion of data only from the Caucasian population in this study, subgroup analysis was not performed. (2) The conclusions of this study still require validation through large-sample, multicenter populations, and functional experiments.

In conclusion, the rs2471738 polymorphism could potentially escalate the risk of AD by modulating the expression of *MAPT* in human brain tissue. However, to substantiate our results, it is imperative to perform further large-scale, multi-center studies and functional analyses.

## Ethics approval and consent to participate

Not applicable.

## Funding

This study was supported by grants from Natural Science Foundation of Anhui Provincial Education Department (Grant No.2022AH050756).

## CRediT authorship contribution statement

**Ning Bin:** Writing – review & editing, Writing – original draft, Conceptualization. **Liu Hongbo:** Formal analysis, Data curation. **Wang Shitao:** Writing – review & editing, Writing – original draft, Conceptualization. **Luo Guoshuai:** Software. **Xu Hui:** Supervision. **Lu Jinghong:** Methodology. **Li Zongyou:** Project administration. **Ding Xiangqian:** Formal analysis. **Sun Guangxin:** Data curation. **Zhang Mengen:** Data curation. **Dong Jingjing:** Resources.

## Declaration of Competing Interest

The authors declare that this work was conducted in the absence of any commercial or financial relationships that could be construed as a potential conflict of interest.
